# Serial ultrasound assessment of diaphragmatic function and clinical outcome in patients with amyotrophic lateral sclerosis

**DOI:** 10.1186/s12890-019-0924-5

**Published:** 2019-08-27

**Authors:** Riccardo Fantini, Roberto Tonelli, Ivana Castaniere, Luca Tabbì, Maria Rosaria Pellegrino, Stefania Cerri, Francesco Livrieri, Francesco Giaroni, Marco Monelli, Valentina Ruggieri, Nicola Fini, Jessica Mandrioli, Enrico Clini, Alessandro Marchioni

**Affiliations:** 1 Respiratory Diseases Unit and Centre for Rare Lung Diseases, Policlinico, University Hospital of Modena, Modena, Italy; 2 Department of Medical and Surgical Sciences, University of Modena Reggio Emilia, Modena, Italy; 3 Clinical and Experimental Medicine PhD Program, University of Modena Reggio Emilia, Modena, Italy; 4 School of Medicine, University of Modena Reggio Emilia, Modena, Italy; 5 Department of Neurosciences, St. Agostino Estense Hospital, Azienda Ospedaliero Universitaria di Modena, Modena, Italy

**Keywords:** Amyotrophic lateral sclerosis, Respiratory failure, Mechanical ventilation, Diaphragm dysfunction, Diaphragm ultrasound

## Abstract

**Background:**

Diaphragmatic assessment by ultrasound (US) is a non-invasive and useful method in the clinical management of patients with Amyotrophic Lateral Sclerosis (ALS). The aim of our observational study was to evaluate the impact of serial assessment of the diaphragmatic function by US on long-term outcomes in a series of patients suffering from ALS and to correlate US indices of diaphragmatic function and respiratory function tests with these outcomes.

**Methods:**

A cohort of 39 consecutive patients has been followed up to 24 months. Both lung volume (forced vital capacity, FVC) and diaphragmatic pressure generating capacity (by sniff inspiratory nasal pressure (SNIP) and by both US thickening fraction, ΔTdi, and the ratio of the thickening fraction between tidal volume and maximal lung capacity, ΔTmax) were recorded at baseline and every 3 months. Parameters were then correlated with outcomes (nocturnal hypoventilation, daily hypercapnia, start of ventilatory support (NIV), and death at 1 year) over time.

**Results:**

The occurrence of ΔTmax > 0.75 increased the risk to start NIV (HR = 5.6, *p* = 0.001) and to die (HR = 3.7, *p* = 0.0001) compared with patients maintaining lower values. Moreover, compared with the occurrence of FVC < 50% of predicted, ΔTmax > 0.75 appeared slightly better correlated with NIV commencement within 6 months.

**Conclusions:**

Serial diaphragmatic assessment by ultrasound is a useful and accurate method to predict the initiation of NIV earlier in patients with ALS.

**Electronic supplementary material:**

The online version of this article (10.1186/s12890-019-0924-5) contains supplementary material, which is available to authorized users.

## Background

Amyotrophic Lateral Sclerosis (ALS) is a progressive neurodegenerative disease involving the motor neurons of the cortex, brainstem and spinal cord. The leading cause of death in these patients is the onset of hypercapnic respiratory failure due to progressive respiratory muscle involvement, including diaphragmatic dysfunction [1].

The early assessment of diaphragmatic function might therefore be essential to prompt non-invasive mechanical ventilation (NIV) with the aim of increasing survival and improving quality of life in these patients [2]. Overall, the early appliance of NIV reduces the progressive loss in forced vital capacity (FVC) and balances the effects of nocturnal hypoventilation [3, 4]. Currently, NIV is recommended when respiratory symptoms begin and lung function tests, namely FVC, decline to suggest failure of the respiratory system [5].

The routinely used evaluations of both lung volumes (namely FVC by spirometry) and diaphragmatic pressure generating capacity (by sniff nasal inspiratory pressure test (SNIP)) require the patient’s full collaboration and coordination, thus very often they are not very informative [6, 7] in those subjects with severe bulbar dysfunction or fronto-temporal dementia. Other neurophysiological tests to assess diaphragmatic function (i.e. phrenic nerve stimulation) can be helpful though uncomfortable and/or difficult to perform routinely [8–10].

Recently, diaphragmatic assessment by ultrasound (US) has been used to distinguish between normal status and dysfunction [11–13] in different clinical settings [14, 15]. This technique requires little patient collaboration unlike the other volitional tests mentioned above [11, 16].

Pinto et al. demonstrated that diaphragm thickness by US correlated with compound motor action potential of the phrenic nerve [17]. Our group showed that US-derived indices of diaphragmatic function (thickening fraction, ΔTdi, and the ratio of the thickening fraction between tidal volume and maximal lung capacity, ΔTmax) correlated with respiratory function tests in patients with ALS [12]. In particular, we have found that ΔTmax is better correlated with diaphragmatic dysfunction than FVC, whose value when lower than 50% of predicted is still recognized as the threshold limit to prompt NIV.

The aim of this study was therefore to evaluate the impact of serial assessment of ΔTmax on long-term outcomes in a series of patients suffering from ALS and to correlate US measurements of diaphragmatic function and respiratory function tests with these outcomes.

## Methods

### Study population

This prospective observational cohort study was carried out at the Respiratory Diseases Unit of the University Hospital of Modena (Italy) over a 36-month period, from January 2013 to January 2016. Patients were recruited from among those referred to the Center of ALS-Neurology Unit of the University Hospital of Modena, with the *possible*, *probable*, *probable laboratory-supported* or *definite* diagnosis of ALS (sporadic or familiar) according to the revised El Escorial criteria [18].

Inclusion criteria for the present study were as follows:

- Age > 18 years.
- Consensus on diagnosis reached < 24 months before enrollment.
- Ability to perform volitional pulmonary function tests (spirometry and sniff test).
- ΔTmax > 0.75 at first visit.

Patients already developing respiratory failure, on NIV regimen or tracheostomy, and those who were pregnant or breastfeeding were excluded.

The Ethics Committee “Comitato Etico dell’Area Vasta Emilia Nord” (Italy) approved the study protocol (registered protocol number 839/C.E.) and written informed consent to participate was obtained from all patients.

### Clinical and functional assessment

Individual clinical (including BMI) and functional data were collected at baseline and every 2–3 months over 2 years.

At baseline, the following information was recorded: family history, gender, age of onset, type of ALS (spinal or bulbar), presence of dementia or extrapyramidal signs, current therapy. In addition, nocturnal standard 4-channel cardiorespiratory monitoring (Embletta PDS™, Flaga hf Medical Devices, Reykjavik, Iceland) was performed at this time to detect hypoventilation.

At each visit during follow-up, patients underwent complete respiratory function (namely pulmonary and diaphragmatic) assessment including spirometry, arterial blood gas analysis, sniff test, and US of the diaphragm by expert technicians blinded to the study purpose.

#### Pulmonary function tests

Spirometry (MasterScreen™ Jaeger, Vyaire Medical Inc., Mettawa, IL, USA) was performed with the patient in the sitting position, and measurements were carried out complying with American Thoracic Society guidelines [19]. The best value out of 3 forced expirations obtained after a maximal inspiration was recorded to determine the FVC.

Before spirometry, a blood sample was taken from the radial artery for analysis of blood gases, while the patient was breathing ambient air at rest; arterial oxygen tension (pO_2_), carbon dioxide tension (pCO_2_), and pH were obtained by an automatic analyzer (ABL90FLEX™, Radiometer Medical ApS, Copenhagen, Denmark). The day before spirometry was performed patients underwent nocturnal oxymetry registration in order to detect the onset of nocturnal hypoventilation.

#### Diaphragmatic function tests

The Sniff test was conducted by inserting a plug in a nostril and connecting it to a pressure transducer (MicroRPM™, CareFusion, Florence, Italy) while the patient performed a maximal inspiratory maneuver through the contralateral nostril. The best SNIP value was recorded among 20 maneuvers starting from functional residual capacity (FRC) and performed every 30 s [16]. FVC > 80% of the predicted value and SNIP test below 70 cmH_2_O in men and 60 cmH_2_O in women were considered to be normal reference values [16, 19].

US examination of the diaphragm was performed while in the sitting position by a system (GE Vivid 7, KPI Healthcare, Yorba Linda, CA, USA) connected to a 7–12 MHz linear probe set to B mode. The probe was positioned to derive the best possible view of the diaphragm searching the point of apposition between the mid-axillary and the posterior-axillary lines. The diaphragm was identified as a three-layered structure consisting of one relatively non-echogenic muscle layer covered by two echogenic lines determined by peritoneal serosa and diaphragmatic pleura. Diaphragm thickness at the end of exhalation was measured in the area of apposition. During US assessment of diaphragm function a spirometer was used to ensure the correlation with the phase of the respiratory cycle. Since the increase in diaphragm thickness during inhalation was used as an indirect measure of muscle contraction, the changes in thickness during tidal volume and after maximal inspiration to total lung capacity (TLC) were also taken [1] (Additional file 1: Figure S1). Measurements were performed three times on both sides of the diaphragm. Images were stored in electronic format and the best out of 3 measurements was recorded for analysis. US measurements were performed just before the patients had undergone respiratory function tests.

Change in diaphragm thickness (Tdi) during inspiration starting from FRC to Vt (so called thickening fraction ΔTdi = [(end-inspiratory Tdi – end-expiratory Tdi) / end-expiratory Tdi] × 100) and the ratio between Tdi at the end of Vt and Tdi after maximal inspiration up to TLC (ΔTmax = end-inspiratory Vt Tdi / end-inspiratory TLC Tdi) were determined. According to previous data, ΔTdi < 20% and ΔTmax > 0.75 were considered thresholds for diaphragmatic dysfunction [12].

### Outcome measures

The association between occurrence of pathological ΔTmax and NIV initiation and mortality were the primary outcomes considered for analysis. The decision to start NIV was taken by a multidisciplinary team unaware of the results obtained by diaphragmatic US assessment and rigorously following the specific criteria set by the American Academy of Neurology with particular regard to respiratory function tests [20]. During the follow-up, the following were also registered: the onset of nocturnal hypoventilation defined as a decrease in hemoglobin saturation below 88% for more than 5 min starting from a hemoglobin saturation level during waking state above 90%, and daily hypercapnia defined as arterial blood pCO_2_ above 45 mmHg [21]. Among patients undergoing NIV treatment, the association between time course of ΔTmax value and mortality was also investigated.

### Statistical analysis

The statistical package GraphPad Prism 7.0 (GraphPad Software, Inc., La Jolla, CA, USA) was used for statistical analysis. A *p* value lower than 0.05 was considered to be statistically significant.

Descriptive statistics were used for demographic, clinical and instrumental variables. Kaplan–Meier curve analysis was used to investigate the impact of ΔTmax > 0.75 or < 0.75 on NIV start and mortality compared to FVC > 50% or < 50%. Linear regression analysis of covariance was applied to ΔTmax time course in the whole sub-group of patients who started NIV and according to their survival within 1 year (survivors and non-survivors). Contingency table analysis for relative risks was used to compare the traditional FVC and SNIP metrics with the proposed new ultrasound guided indices (ΔTmax and ΔTdi) in predicting the onset of recorded clinical outcome measures (hypercapnia, nocturnal hypoventilation, NIV start, and death).

## Results

**Fig. 1:**
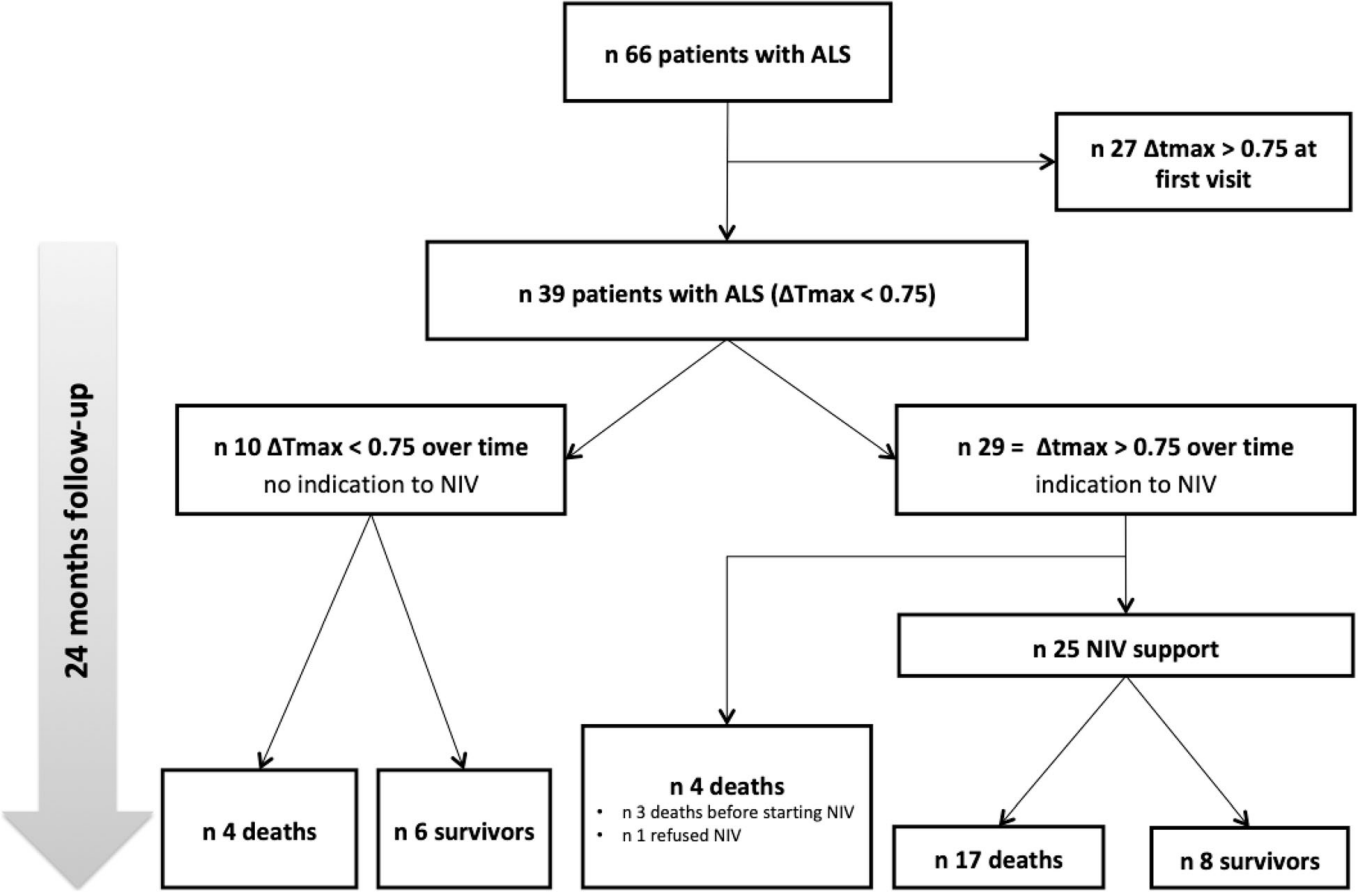
Flow diagram for the patients in this study

In total, 66 consecutive outpatients with ALS were referred to our Unit. Out of these, 39 subjects fulfilled entry criteria and were enrolled in the study; 27 already presented an abnormal ΔTmax (> 0.75) and were therefore excluded (see Fig. 1).

The general characteristics and clinical features of the population at study baseline are presented in Table 1. Of note, 12 patients (31%) presented a bulbar onset of their disease. At that time, US indices ΔTmax and ΔTdi and respiratory function variables FVC and SNIP were not significantly different between patients with bulbar or spinal involvement.

**Table 1:** Characteristics of the study population at baseline. Data are presented as number/percentage for dichotomous values and mean value with interquartile range for continuous values

Parameter	Value
Patients (n)	39
Male (n/%)	27 (69)
Age (years)	65.9 (57–74)
BMI	25.3 (21.2–27.8)
Bulbar onset (n/%)	12 (31)
Age at diagnosis (years)	65 (56–71)
Time to death from ALS diagnosis (months)	41.1 (12.9–68.1)
FVC (% predicted)	82.4 (60.5–103)
Arterial paCO_2_ (mmHg)	40.9 (37.6–43.4)
SNIP (cmH_2_O)	50 (39–66.5)
US Δ*T*_max_	0.60 (0.52–0.69)
US Δ*T*_di_ *V*_t_ (%)	51 (28.4–70.4)
Follow-up duration (months)	14 (6–24)

Legend: *FVC* Forced Vital Capacity, *paCO*_*2*_ partial arterial carbon dioxide, *SNIP* Sniff Inspiratory Nasal Pressure, *US* Ultrasound, *ΔT*_*max*_ ratio between diaphragm thickness at the end of tidal volume and after maximal inspiration, *ΔT*_*di*_ *V*_*t*_ change in diaphragm thickness during spontaneous breathing at tidal volume

Ten out of the 39 patients (26%) had normal ΔTmax values during the follow-up (Fig. 1). Among these, 4 died due to causes unrelated to ALS-induced ventilatory failure (2 ischemic heart disease, and 2 sepsis). Out of the 29 patients who shifted to an abnormal ΔTmax, 25 received NIV, while 3 died before receiving NIV, and 1 refused treatment. On average, an abnormal ΔTmax value preceded the decision to start NIV by 2.8 months (IQR 0.4–5.4 months) compared with criteria based on assessment with respiratory function tests. The time from NIV start to death was 8.9 months (IQR 3.1–15.8 months).

**Fig. 2:**
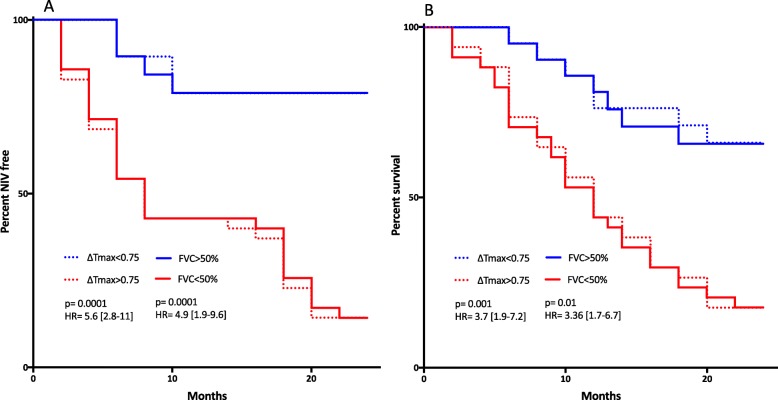
Kaplan–Meyer curves for percentage of patients NIV-free (panel **a**) and percentage survival (panel **b**) during follow-up according to their normal (Δ*T*_max_ *< 0.75*) or abnormal (Δ*T*_max_ *> 0.75*) threshold levels of diaphragmatic dysfunction as assessed by the ultrasound monitoring technique

**Fig. 3:**
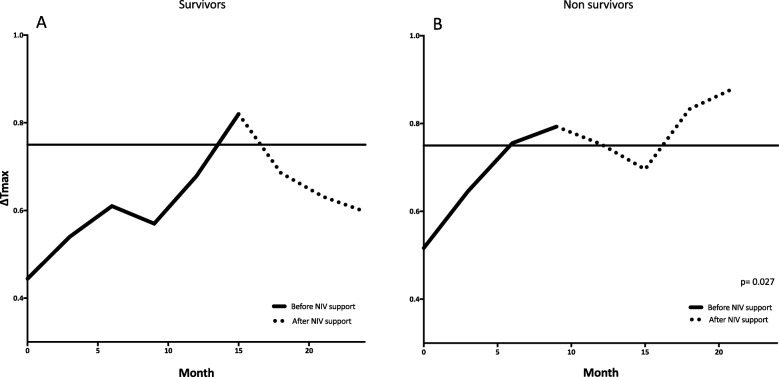
Average time course of Δ*T*_max_ as recorded by the ultrasound technique in Survivors (panel **a**) and Non-survivors (panel **b**) undergoing NIV. Continuous line indicates the period under unassisted breathing, dotted portion shows the period following NIV commencement

Figure 2 presents the Kaplan–Meier curves of both NIV-free and survival rates in groups with normal (< 0.75) or abnormal (> 0.75) ΔTmax as recorded over time compared to FVC values below or above 50%; the risk to start NIV (HR = 5.6, *p* = 0.001) and to die (HR = 3.7, *p* = 0.0001) was several-fold increased in patients with ΔTmax > 0.75 and/or with FVC below 50% compared with others. In addition, change over time of average ΔTmax in the 25 patients who started NIV and according to survival is shown Fig. 3; the trend of ΔTmax clearly and significantly differed (*p* = 0.027) between patients who survived or died following NIV.

Contingency table analysis for relative risks of respiratory function (FVC, SNIP) and US (ΔTmax, ΔTdi) variables in predicting the onset of the outcomes considered is summarized in Table 2. Notably, the occurrence of FVC < 50% of predicted and the presence of ΔTmax > 0.75 in the follow-up period, share similar relative risks for starting NIV within 6 months and death within 12 months.

**Table 2:** Contingency table analysis of risks for clinical outcomes among the functional indices as recorded in the study patients. Data are presented as relative risk [95% CI] (*p* value)

Variable	Outcome			
	Hypercapnia	Nocturnal hypoventilation	NIV start within 6 months	Death within 12 months
Δ*T*_max_ > 0.75	3.3 [1.14–6.72] (0.01)	3.11 [1.03–8.64] (0.03)	2.1 [1.09–4.5] (0.01)	2.5 [1.15–5.4] (0.02)
Δ*T*_di_ *V*_t_ < 20%	1.7 [1.02–3.4] (0.04)	2 [1.03–4-44] (0.01)	2.0 [1.08–5.3] (0.03)	2.6 [0.87–5.4] (0.2)
SNIP < 40 cmH_2_O	4.2 [1.9–6.9] (< 0.001)	2.9 [1.05–4.9] (0.005)	1.7 [1.3–5.5] (0.001)	1.6 [1.16–6.5] (0.04)
FVC < 50%	3.2 [1.2–7] (0.006)	3 [1.1–7.5] (0.01)	1.8 [1.2–3.4] (0.01)	2.2 [1.04–6.4] (0.03)

*Legend*: *ΔT*_*max*_ ratio between diaphragm thickness at the end of tidal volume and after maximal inspiration, *ΔT*_*di*_ *V*_*t*_ change in diaphragm thickness during spontaneous breathing at tidal volume, *SNIP* Sniff Inspiratory Nasal Pressure, *FVC* Forced Vital Capacity

## Discussion

With this prospective observational study, we have demonstrated that serial diaphragmatic assessment by ultrasound may be useful to predict initiation of NIV and death in patients with ALS. The occurrence of an abnormal ΔTmax value over time preceded the decision to start NIV.

From technical point of view, the site of US exploration of the diaphragm changes among studies. For example Pinto et al. [8] used the space between the mid axillary and antero-axillary lines while Carriè [22] et al. explored the area between the mid clavicular and anterior axillary line. In our study in order to perform highly accurate US evaluation of the diaphragm, the probe was positioned to explore the point of apposition between the mid-axillary and the posterior-axillary lines. We decided to choose the point of apposition as to detect the thicker part of the muscle in order to minimize possible measurements errors.

A ΔTmax value > 0.75 has previously been demonstrated by our group as the threshold limit of normal diaphragmatic function in patients with ALS [12]. In particular, we were able to show that ΔTmax > 0.75 presented a 75% sensitivity and 85% specificity in identifying subjects with FVC values lower than 50% of the predicted value. To date, FVC is still considered the most valid measure to decide when to apply NIV and to manage respiratory failure in these patients [20]. Quite interestingly, we provide new information suggesting that deterioration of ΔTmax, although at a risk similar to the behavior of FVC, precedes the occurrence of a threshold limit to prompt NIV by around 3 months.

In addition, the occurrence of abnormal ΔTmax over time correlates with mortality similar to the occurrence of abnormal FVC, still the best respiratory prognostic indicator of survival and disease progression in ALS [23]. Moreover, since we were able to demonstrate that the occurrence of an abnormal ΔTmax in a patient is a risk factor for the development of daily hypercapnia and nocturnal hypoventilation, these findings taken together strengthen the potential role of the noninvasive US assessment of the diaphragm in this clinical setting.

In our study, we have shown that the occurrence of an abnormal thickening fraction of the diaphragm (ΔTdi < 20%) [15] is a significant risk factor for the onset of daily hypercapnia and nocturnal hypoventilation and the need for non-invasive mechanical ventilation within 6 months (see Table 2). However, we did not find a significant relationship between ΔTdi < 20% and death in the following 12 months. Though this finding might seem contradictory if we consider ΔTdi to be a late index of diaphragmatic dysfunction (see discussion which follows), it could be explained by the small number of patients developing ΔTdi < 20% (*n* = 9). Therefore, though ΔTmax and ΔTdi, as a surrogate measure of diaphragmatic pressure generating capacity, might seem similar in describing diaphragmatic function, they could provide different information on muscle status.

When the change in diaphragm thickness (ΔTdi) in the area of apposition is lower than 20%, diaphragmatic dysfunction should be considered very severe and close to paralysis [13]. As such, this finding is certainly useful; however, it only allows identification of the late phase of dysfunction, but not an early stage of muscle fatigue and/or weakness. *Muscle fatigue* can be defined as a condition characterized by a reduction in muscle capacity to generate force when subjected to a given load. This condition is reversible following rest for a certain period of time, and it is different from the concept of *muscle weakness* where the ability to restore strength after rest is compromised [24, 25].

In neuromuscular diseases, the elastic load of the respiratory system increases due to different factors causing the reduction in lung distensibility: 1) loss of gas-containing alveoli (patchy atelectasis), 2) generalized increase in surface tension of the alveolar lining layer caused by breathing at low volume, 3) alteration in lung tissue elasticity [26]. Therefore in patients suffering from ALS, the increase in elastic load to which the respiratory muscles are subjected (with the consequent increase in transdiaphragmatic pressure [Pdi]), and the reduction in inspiratory muscle force (reduction in maximal Pdi), lead to a Pdi/Pdimax ratio which predisposes to the development of diaphragmatic fatigue.

With this scenario, the non-invasively obtained ΔTmax, as a surrogate measure of Pdi/Pdimax ratio, could be used to provide specific information about the fatigue status of the diaphragm in patients with ALS. Despite this assumption presenting obvious limitations, it is possible to deduce that, when the thickness of the diaphragm at the end of the tidal breath approaches the thickness at TLC, we are probably close to the area of “fatigue/weakness” of the muscle [25].

In our study the average starting point for ΔT max was worse for the non-survivor group but the two values did not differ significantly (0.44 VS .51, *p* = 0.32). We thus believe that the two groups may be considered homogeneous at the beginning of assessment. Patients treated with long-term ventilator support and with better prognosis showed a stabilization of ΔTmax over time following the beginning of NIV (see Fig. 3). This could mean that, in these patients, diaphragmatic function was in the “fatigue area” with muscle rest provided by NIV able to improve performance, whereas in those who died under NIV, the progressive deterioration of ΔTmax is likely to suggest that their diaphragm was in a “weakness zone” which received no benefit from rest (NIV).

If confirmed on larger populations and/or in comparison with other physiological indices, this information on the status of the diaphragm as obtained non-invasively by the US technique could be extremely important in identifying the subset of patients with ALS at greater benefit from early ventilatory support.

Notwithstanding, the present findings only provide preliminary information and at best open a new avenue. Indeed, the lack of comparison between the US assessment of diaphragmatic function and other methods used to assess its contractility [16] still remains a major limitation. Moreover our study lacks from data on intra- and inter-rater variability for the US measurements performed.

## Conclusions

This study suggests that serial diaphragmatic assessment by ultrasound may help identifying patients who require NIV earlier. Further studies to demonstrate correlation with other clinical markers and clinical outcomes are needed.

## Additional file

Additional file 1: **Figure S1.** Correlation between lung volume and thickness of the diaphragm assessed by ultrasound technique. The arrows indicate the thickness of the diaphragm at Total Lung Capacity (TLC), Tidal Volume (Vt), Functional Residual Capacity (FRC). (PNG 508 kb)

## Data Availability

The datasets used and/or analysed during the current study are available from the corresponding author on reasonable request.

## Abbreviations

ALS: Amyotrophic Lateral Sclerosis
FVC: Forced vital capacity
HR: Hazard ratio
NIV: non-invasive mechanical ventilation
PaCO_2_: arterial carbon dioxide pressure
Pdi: transdiaphragmatic pressure
SatO_2_: oxygen saturation
SNIP: Sniff Nasal Inspiratory Pressure
Tdi: diaphragm thickness
TLC: Total lung capacity
US: ultrasound
Vt: tidal volume
