# Supplementary figures and images for: Serial ultrasound assessment of diaphragmatic function and clinical outcome in patients with amyotrophic lateral sclerosis
Source: BMC Pulm Med. 2019 Aug 27;19:160. doi: 10.1186/s12890-019-0924-5 (PMC6712740; doi:10.1186/s12890-019-0924-5)

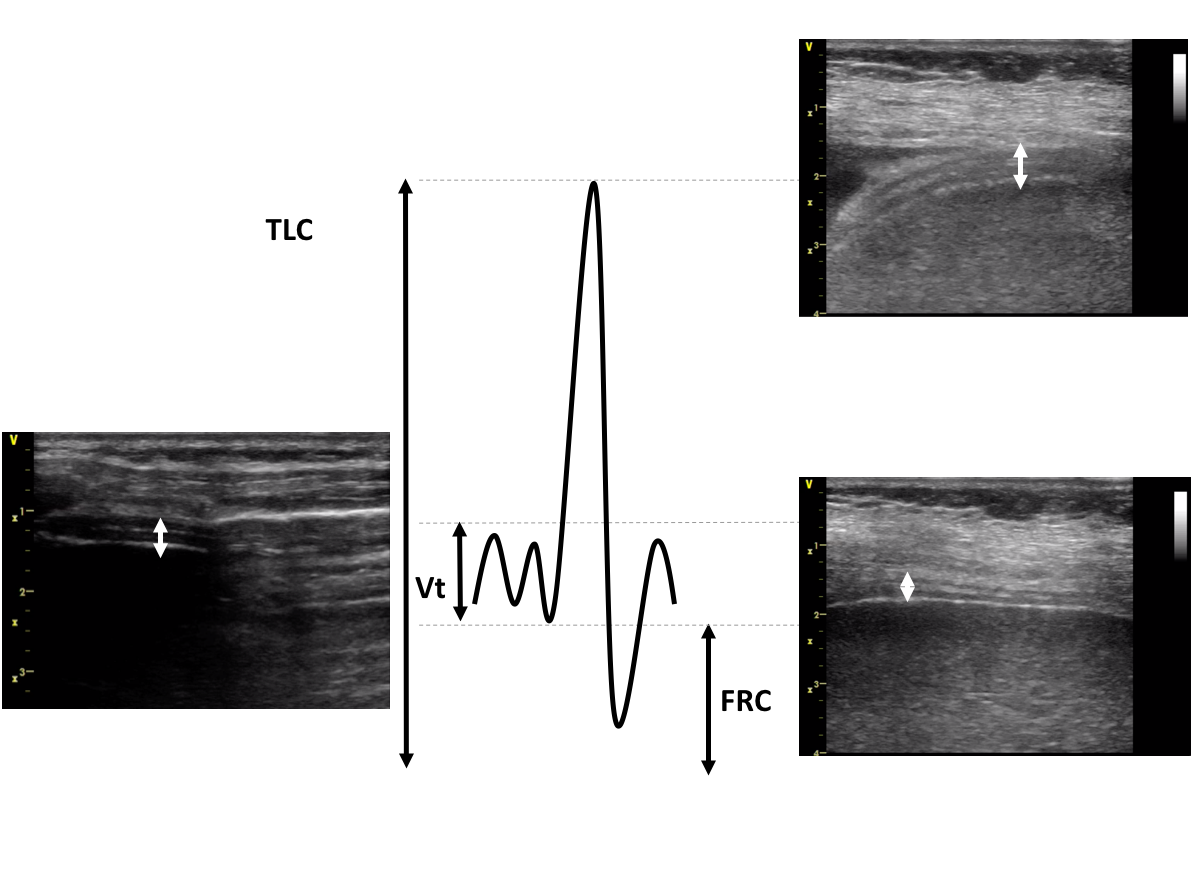

Supplement: Supplementary file 1 — Figure S1. Correlation between lung volume and thickness of the diaphragm assessed by ultrasound technique. The arrows indicate the thickness of the diaphragm at Total Lung Capacity (TLC), Tidal Volume (Vt), Functional Residual Capacity (FRC). (PNG 508 kb) [file 12890_2019_924_MOESM1_ESM.png]
